# A numerical study on the closed packed array of gold discs as an efficient dual mode plasmonic tweezers

**DOI:** 10.1038/s41598-021-99633-x

**Published:** 2021-10-19

**Authors:** Abolfazl Aqhili, Sara Darbari

**Affiliations:** Nano Plasmo-Photonics Research Group, Faculty of ECE, Tarbiat Modares University, 14115-111 Tehran, Iran

**Keywords:** Optical manipulation and tweezers, Optics and photonics, Optofluidics

## Abstract

In this report, we propose the closed pack array of gold discs on glass, as a dual mode plasmonic tweezers that benefits from two trapping modes. The first trapping mode is based on leaky surface plasmon mode (LSPM) on the gold discs with a longer penetration depth in the water and a longer spatial trapping range, so that target nanoparticles with a radius of 100 nm can be attracted toward the gold surface from a vertical distance of about 2 µm. This trapping mode can help to overcome the inherent short range trapping challenge in the plasmonic tweezers. The second trapping mode is based on the dimer surface plasmonic mode (DSPM) in the nano-slits between the neighboring gold discs, leading to isolated and strong trapping sites for nanoparticles smaller than 34 nm. The proposed plasmonic tweezers can be excited in both LSPM and DSPM modes by switching the incident wavelength, resulting in promising and complementary functionalities. In the proposed plasmonic tweezers, we can attract the target particles towards the gold surface by LSPM gradient force, and trap them within a wide half *width* *half* *maximum* (HWHM) that allows studying the interactions between the trapped particles, due to their spatial proximity. Then, by switching to the DSPM trapping mode, we can rearrange the particles in a periodic pattern of isolated and stiff traps. The proposed plasmonic structure and the presented study opens a new insight for realizing efficient, dual-mode tweezers with complementary characteristics, suitable for manipulation of nanoparticles. Our thermal simulations demonstrate that the thermal-induced forces does not interefe with the proposed plasmonic tweezing.

## Introduction

In 1970, the far field optical trapping of particles was introduced by Ashkin^1^ for the first time. Optical trapping has been attractive for applications in biology, physics, and environmental sciences, due to possibility of accurate, contactless, and non-destructive manipulation of small target particles. However, due to diffraction limit, optical trapping suffers from the challenge of trapping particles with sub-wavelength dimensions. Plasmonic tweezers, benefiting from inherently sub-wavelength plasmonic field confinement have been proposed to overcome this challenge. Different plasmonic tweezers have been reported based on localized surface plasmons (LSPs), and surface plasmon polaritons (SPPs). Different LSP structures, including nano-hole with a built-in light source^2^, double nano-holes^3^, nano-triangles^4^, and gold cauldrons^5^ have been utilized for plasmonic tweezers, owing to their highly confined plasmonic fields at the sharp edges. However, having very low penetration depth in the dielectric, LSPs have a low spatial range of trapping^6^, which generally requires an additional technique to guide the target particles into the plasmonic structures in the channel. Regarding this, there are experimental reports that have utilized opto-thermal-induced flow^7^, optical tweezers^4,5^, and integrated electrostatic cells^8^ to attract the target particles to the vicinity of the plasmonic structures, wherein they can be trapped by plasmonic forces. Furthermore, there are other reports that introduce a microfluidic flow, termed as the electro-thermo-plasmonic (ETP) flow, to capture suspended particles and deliver them towards the illuminated plasmonic nanostructures^9,10^. On the other hand, SPPs in structures such as gold strips^11,12^, graphene sheets^13^, graphene strips^14^, and graphene nano-ring resonators^15^ have been used for sorting and moving nanoparticles. SPP fields present greater penetration depth in the dielectric medium in comparison with LSPs^11,12^. Thus, SPP-based tweezers have a higher spatial trapping range, and can attract the particles from further distances in the fluidic channel. Nevertheless, SPPs are mostly used for sorting target particles by deflecting them from the straight fluidic flow, and pushing them toward the appropriate outlet^11,12^. Otherwise, interference patterns of counter propagating SPPs can be used to trap the particles at specific positions, which can be tuned by the input incident powers^16^.

Introducing a plasmonic tweezers with the ability to excite both the LSPs and SPPs modes, we can benefit from the advantages of both plasmonic modes, while overcoming the related drawbacks. Here, we propose a new gold plasmonic structure, consisting of a closed pack array of gold nano-discs to trap target nanoparticles by exciting both the LSPs and SPPs. By exciting SPPs, and allowing appropriate edge reflections in gold discs, we can achieve standing surface plasmonic waves, known as the leaky surface plasmon modes (LSPMs)^17,18^. We will show that plasmonic tweezers based on the achieved LSPMs can lead to a high trapping spatial range, while it is capable of fixing the particles at certain positions. On the other hand, due to the nanometer spacing between the closed pack nano-discs array, plasmonic cavity modes can be excited and dimer surface plasmon mode (DSPM) trapping can be utilized in the same gold plasmonic structure. DSPMs are inherently highly spatial confined plasmonic modes, which can lead to high plasmonic gradient forces, and completely isolated plasmonic trapping sites, so that single quantum dots can be trapped separately. Taking advantage of both LSPMs and DSPMs in a plasmonic tweezers, we can attract quantum dots from micrometer-scale distances towards the plasmonic structure by LSPMs, then by switching the incident wavelength we can strongly trap the small particles by DSPMs. This dual mode plasmonic tweezing operation can allow investigation of interactions between the nanoparticles. The presented plasmonic tweezers can be used to realize efficient lab-on-a-chip systems, benefiting from dual tweezing modes with complementary trapping properties, for the first time. We design the plasmonic structure, so that the DSPM and the LSPM leaky plasmonic modes are excitable in a single plasmonic structure, at the excitation wavelengths that are compatible with plasmonic tweezing commercial configurations, and aqua environment in microfluidic channels. We also present a systematic study on the plasmonic physics and characteristic of a single and the closed packed array of gold discs to achieve an appropriate insight for designing desirable plasmonic tweezing behaviors. Moreover, the proposed structure benefits from a simple and low cost fabrication process, which is proposed to be implemented by using microsphere lithography. In the following sections, we thoroughly investigate and compare the mentioned two plasmonic modes in the proposed structure, then utilize these modes for particle trapping.

## Proposed structure and operation principle

### The proposed structure

**Figure 1:**
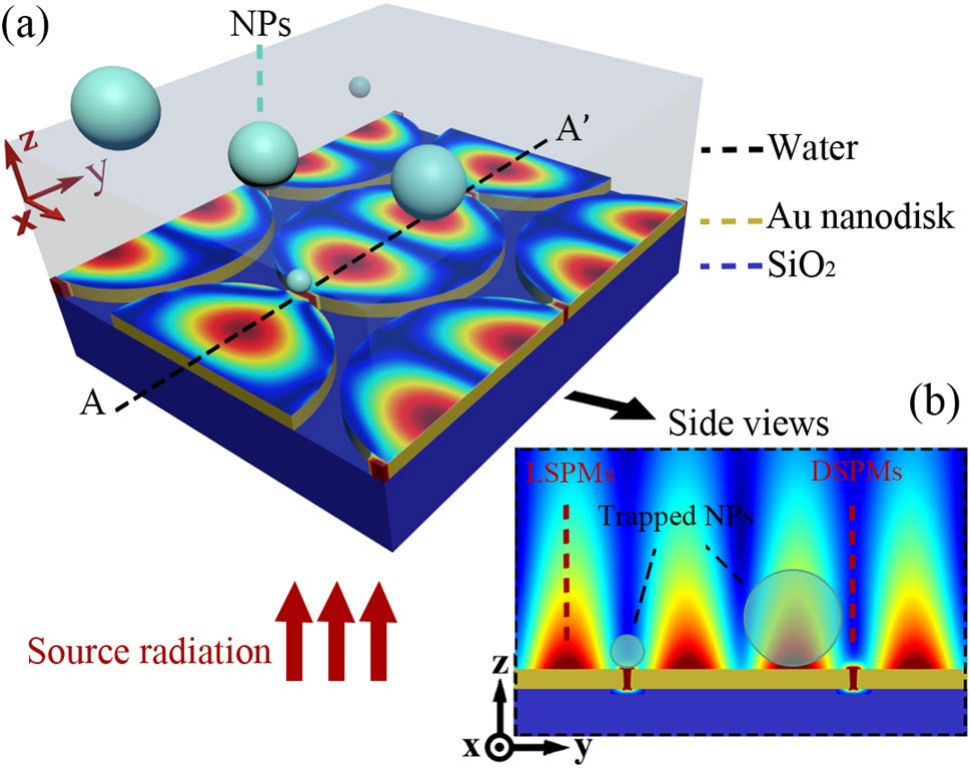
(**a**) Three-dimensional, and (**b**) side view schemes of the proposed closed pack array of gold nano-discs with a qualitative representation of the excited plasmonic fields.

Figure 1a shows the schematic top view of our proposed periodic structure, which consists of an array of gold nano-discs with radius of 300 nm, gold thickness of 40 nm, and interspacing gap of 10 nm over a SiO_2_ substrate. Moreover, this figure shows the qualitative field distribution in the *x*–*y* plane on top of the gold surface of the array, which is illuminated by a *y*-polarized normal incident plane wave. Figure 1b manifests the cross section of the structure along the dashed black line (AA’) in part (a). This figure elucidates that the spacing between the neighboring gold discs forms nanometric slits, and can lead to excitation of highly confined dimer surface plasmon modes (DSPMs), which can serve as strong plasmonic trapping sites. These DSPMs show high plasmonic field confinement and low penetration depth, and can lead to efficient plasmonic trapping of nanometric target particles smaller than about 34 nm, at the vicinity of nano-slits. Moreover, Fig. 1b indicates that the proposed periodic disc array can behave as a metallic grating and lead to excitement of surface plasmons (SPs) at the gold/water interface. The excited SPs on the discs can reflect from the disc edges and lead to formation of standing SP modes on the discs, leaking to the water medium with a high penetration depth in comparison with DSPMs. These leaky surface plasmon modes are defined as LSPMs, and can lead to plasmonic trapping sites with a larger spatial range in the vertical direction, but weaker trapping potential depth for trapping small particles (< 34 nm), comparing with DSPMs. It is well known that most plasmonic tweezers suffer from the short range behavior of the resulted plasmonic fields, which extensively confines their affecting vertical distance along the microfluidic channels, and can limit the operation efficiency of the designed optophoresis system. Hence, we have taken advantage of the described LSPMs in the presented plasmonic structure, to trap or attract the target particles toward the disc surfaces within a larger vertical distance from the discs surface. Regarding this, the designed plasmonic tweezers does not need any additional mechanisms^19–21^ to bring the target particles in the vicinity of the plasmonic structures.

Therefore, in the presented structure we are able to excite two different plasmonic modes (DSPMs and LSPMs) with different trapping characteristics, by switching the incident wavelength. In other words, we can excite LSPMs to attract the target particles beyond micrometer vertical distance from the metal surface, and then switch the incident wavelength to excite DSPM trapping, in order to achieve a strong and stable trapping operation. Furthermore, because the DSPMs field and their trapping sites are spatially confined and isolated, they lead to isolated trapped particles, as compared with the LSPMs trapping. LSPM trapping leads to closer trapping sites, which can be suitable for studying the interaction between the target nanoparticles. As a result, our proposed system allows different trapping functionalities, and can be proposed to design new generations of long range and strong plasmonic trapping of nanometer target particles, which is attractive for lab-on-a-chip applications.

### The modal behavior of LSPMs

First, we analyze the excitement of SPPs at the gold/dielectric interfaces of the discs. Solving Maxwell's equations at a flat interface of metal/dielectric, the plasmon wave vector along the y direction can be approximated from the SPPs dispersion relation of ${\mathrm{k}}_{\mathrm{SP}}=2\uppi /{\uplambda }_{0}\times ({{\varepsilon }_{\mathrm{m}}{ \varepsilon }_{\mathrm{d}}/({\varepsilon }_{\mathrm{m}}+{\varepsilon }_{\mathrm{d}} ))}^{0.5} {\widehat{\mathrm{ a}}}_{\mathrm{y}}$, wherein ${\lambda }_{0}$ is the wavelength of source, ${\varepsilon }_{m}$, and ${\varepsilon }_{d}$ are the relative permittivities of the metal and the dielectric layers, and ${\widehat{\mathrm{a}}}_{y}$ is the unitary vector along the y direction^22^. On the other hand, it is well known that ${\mathrm{k}}_{\mathrm{SP}}$ is much larger than the light wave vector in the dielectric medium (${\mathrm{k}}_{\mathrm{d}}=2\uppi /{\uplambda }_{0}\times {{\varepsilon }_{\mathrm{d}}}^{0.5}$). Due to this significant wave vector mismatch, SPPs at the metal/dielectric interface cannot be simply excited. Creating metallic gratings^12,23,24^ at the metal/dielectric interface is one of the common approaches to overcome this mismatch, and to excite SPPs at the metal surface. The grating wave vector is calculated from ${\mathrm{k}}_{\mathrm{G}}={\mathrm{k}}_{\mathrm{G},\mathrm{x}}{\widehat{ a}}_{x}+{\mathrm{k}}_{\mathrm{G},\mathrm{y}} {\widehat{a}}_{y}$, wherein ${\mathrm{k}}_{\mathrm{G},\mathrm{x}}=\mathrm{N}\times 2\uppi /{\Lambda }_{x}{\widehat{ a}}_{x}$, and ${\mathrm{k}}_{\mathrm{G},\mathrm{y}}=\mathrm{M}\times 2\uppi {/\Lambda }_{y} {\widehat{a}}_{y}$ are the wavevector components along *x* and *y* directions. *Λ*_*x*_ and *Λ*_*y*_ are the grating periods along *x* and *y* directions, respectively. *N* and *M* are integers that represent the number of plasmon wavelength in one period of *x* and *y* directions, respectively. Thus, to excite the SPs in the grating configuration, the incident wavelength (*λ*_*0*_) should satisfy k_SP_ = k_G_. In our proposed structure, due to the linear polarization of the source along the *y* direction, ${\mathrm{k}}_{\mathrm{G},\mathrm{x}}$ can be neglected. Thus, the excitation wavelength of LSPMs on the gold discs of our proposed structure can be approximated as^25^:

1 $${\lambda }_{{SP}_{M,j}}=\frac{{\Lambda }_{y}}{M}({\frac{{{n}_{m}}^{2}{{n}_{j}}^{2}}{{{n}_{m}}^{2}+{{n}_{j}}^{2}})}^\frac{1}{2}; j=a,s \, M=1, 2, \dots $$

wherein $a,s,$ and *m* stand for the ambient (water), the substrate (SiO_2_), and the metal (gold), and n is the refractive index. Noting that there are two different metal/dielectric interfaces in our proposed structures (the interfaces of gold discs with water and SiO_2_), there are two LSPM modes, which are represented by plasmon wavelengths of ${\lambda }_{{SP}_{M,j}}$($\mathrm{j}=\mathrm{a},\mathrm{s}$).

### The modal behavior of DSPMs

It is known that for a pair of metallic nanoparticles (known as dimers) with very small spacing, when LSPs are excited by light illumination, the interaction between the excited dipoles of each nanoparticle becomes significant^26,27^. This interaction will modify the total optical response and produce coupled modes with very strong fields in the spacing gaps between the metal nanoparticles, which are called DSPMs, here^28^. Depending on the spacing gap size between the metal nanoparticles, quadrapole or octapole modes can also be excited in addition to the dipole mode in DSPMs^29^. It has been proved theoretically and experimentally that the intensity and distribution of the excited dimer fields depend on the shape, size, and the orientation of the metallic nanoparticles^26,30^. The effective interaction of the nanoparticles in the dimer mode depends on the alignment of the linear polarization direction of the source with respect to the dimer axis^31,32^. Among metallic plasmonic structures, gold nano-discs have shown promising properties of DSPMs^33^. Furthermore, the periodic arrangement of metal nano-discs has been successfully used in various applications such as the surface-enhanced Raman scattering (SERS), and high performance LSPR sensors, and surface lattice resonances (SLR)^34,35^.

Implementation of DSPMs have been realized by using complicated and high resolution fabrication techniques such as electron beam lithography (EBL), ion beam milling and stamp-printing, or combination of etch-back and template stripping technique^36,37^. Here, we propose a closed pack gold disc array to achieve DSPMs, which can be realized by combination of micro/nano sphere lithography and lift-off technique, as a low cost fabrication technique. In this report, we numerically investigate the plasmonic modes, and the relating trapping behavior of the proposed closed pack gold disc array.

### Simulation method

We have employed 3D finite difference time domain (3D-FDTD)^38^ method to numerically solve the Maxwell’s equations in our proposed structure. Then, the time-averaged net plasmonic force exerted to the mass center of the target particles are determined by calculating the surface integral of the time-averaged Maxwell Stress Tensor (T)^39,40^:

2 $$T\left(r,t\right)=\varepsilon E\left(r\right){\otimes E}^{*}\left(r\right)+\mu H\left(r\right)\otimes {H}^{*}\left(r\right)-\frac{1}{2}({\varepsilon \left|E\left(r\right)\right|}^{2}+{\mu \left|H\left(r\right)\right|}^{2})$$

3 $$\langle F\rangle =\frac{1}{2}Re{\oint }_{\Omega }^{ }T\left(r,t\right).\widehat{n }ds$$

wherein, **r**, *t*, **n**, *ε,* and *μ* are the position vector, time, the unitary normal vector of surface *S,* that encloses volume Ω around the particle, the permittivity, and the permeability of the surrounding medium, respectively. Obtaining the net optical force, the resulting optical potential energy (*P.E.*) can be derived as:

4 $$P.E.\left(r\right)=-{\int }_{\infty }^{r}\langle F\left({r}^{^{\prime}}\right)\rangle dr{^{\prime}}$$

Moreover, for a trapped particle, the plasmonic force exerted to the particle is linearly related to the displacement of the particle around its equilibrium trapping point. Therefore, the plasmonic trapping stiffness in the *y* direction (*s*_*y*_) can be calculated by^6,41^:

5 $${s}_{y}=-\frac{{\partial F}_{y}}{\partial y}| Equilibrium \, Point$$

## Results and discussions

### Investigation and comparison of LSPMs and DSPMs

**Figure 2:**
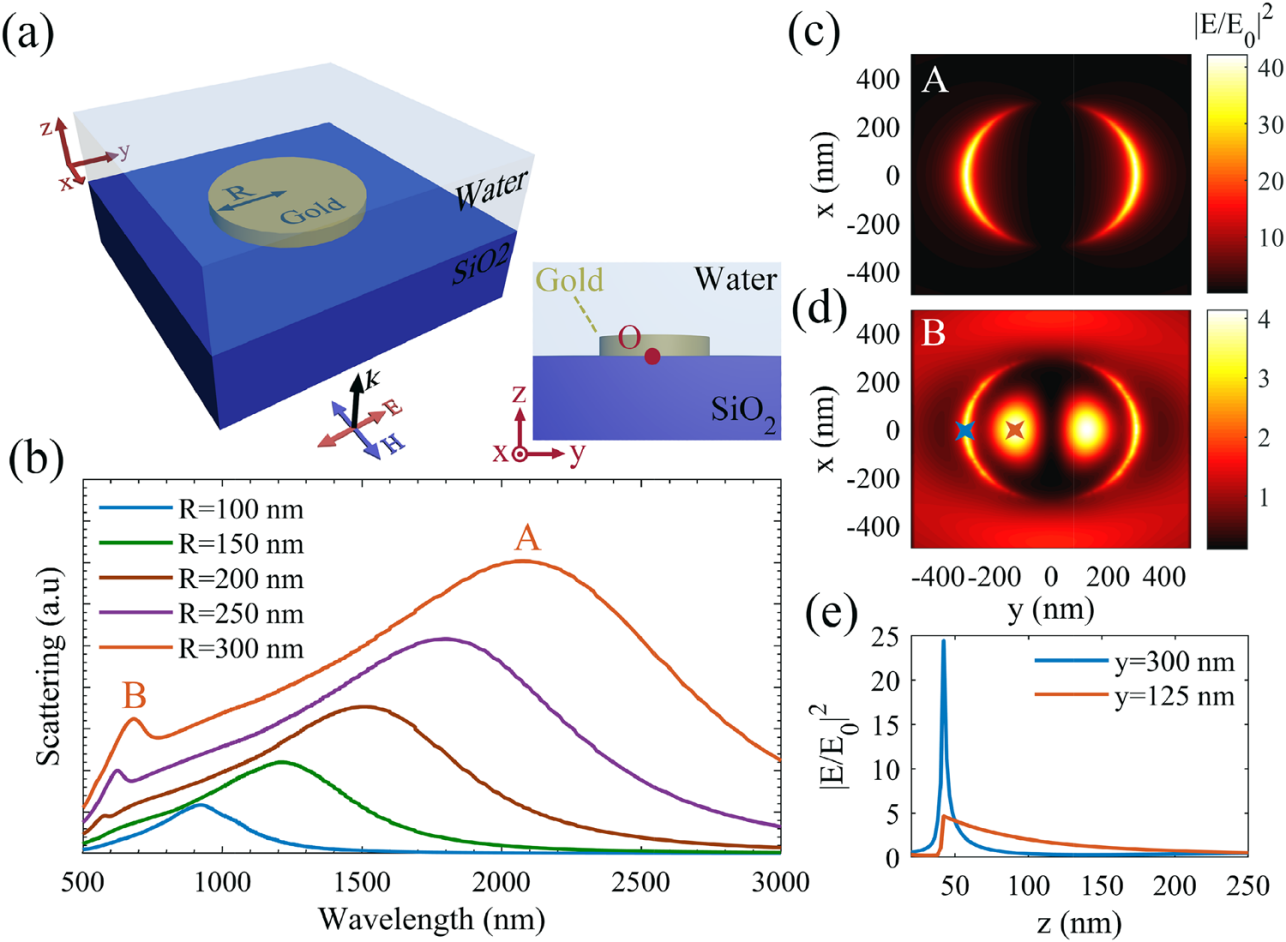
(**a**) Scheme of a single gold nano-disc, which is shown illuminated by a *y*-polarized input source. (**b**) The calculated scattering spectra of the nano-disc with *R* = 100, 150 nm, 200 nm, 250 nm, and 300 nm. The top view normalized field intensity distributions at *z* = 50 nm for (**c**) *λ*_*0*_ = 2080 nm (marked by A), and (**d**) *λ*_*0*_ = 680 nm (marked by B). (**e**) The normalized plasmonic field intensity along the normal directions at *x* = 300 nm, y = 0 (marked by the blue cross in part (**d**)), and at *x* = 125 nm, *y* = 0 (marked by the red cross in part (**d**)).

To investigate the plasmonic properties of the proposed structure, first we study a single gold disc, and calculate the relating near-field scattering spectrum. The investigated gold disc is on a SiO_2_ substrate, with a gold thickness of 40 nm and radius of *R*, and is illuminated normally by a *y*-polarized laser beam (Fig. 2a). The origin of the coordinates is assumed at the center of the gold disc, as shown by a red dot in the side view scheme in part (a). The achieved scattering spectra for discs with radius of *R* = 100, 150, 200, 250, 300 nm are plotted in Fig. 2b. The scattering peaks, marked by A in this figure, indicate the first plasmonic modes, while the second plasmonic modes are emerged in the scattering spectrum for *R* > 200 nm and are marked by B. Figure 2c,d show the top view normalized field distribution at *z* = 50 nm (10 nm above the disc surface) for *R* = 300 nm, corresponding to the plasmonic peaks of A and B in Fig. 2b, respectively. It is observable that modes A are related to the plasmonic field excited at disc edges, while modes B are related to the LSPMs on the gold discs. The observed LSPMs are attributed to the standing SPs, which are originated from the interference of reflected SPPs from the disc edges, when the disc diameter becomes comparable with a factor of incident wavelength^16^. Figure 2e indicates the variations of the normalized field intensity along the *z* direction at *x* = 0 nm, for *y* = 125 nm (red curve) and *y* = 300 nm (blue curve), corresponding to the *x*–*y* position of red and blue cross marks in part (d), respectively. It can be observed that LSPMs show lower field intensities than edge modes in the vicinity of gold surface (low *z* values), but their penetration depth along the *z* direction is significantly larger than the edge modes. In other words, edge plasmonic modes are highly confined along the *z* direction, leading to high plasmonic gradient force with a short range behavior along the *z* direction. In contrary, LSPMs can lead to low plasmonic gradient force, but long range behavior, allowing attraction of target particles from a longer distance. Regarding this, the proposed plasmonic tweezers configuration can open new horizons to overcome the short range behavior drawback of the conventional plasmonic tweezers, while can also benefit from the strong trapping behavior by switching the incident wavelength.

**Figure 3:**
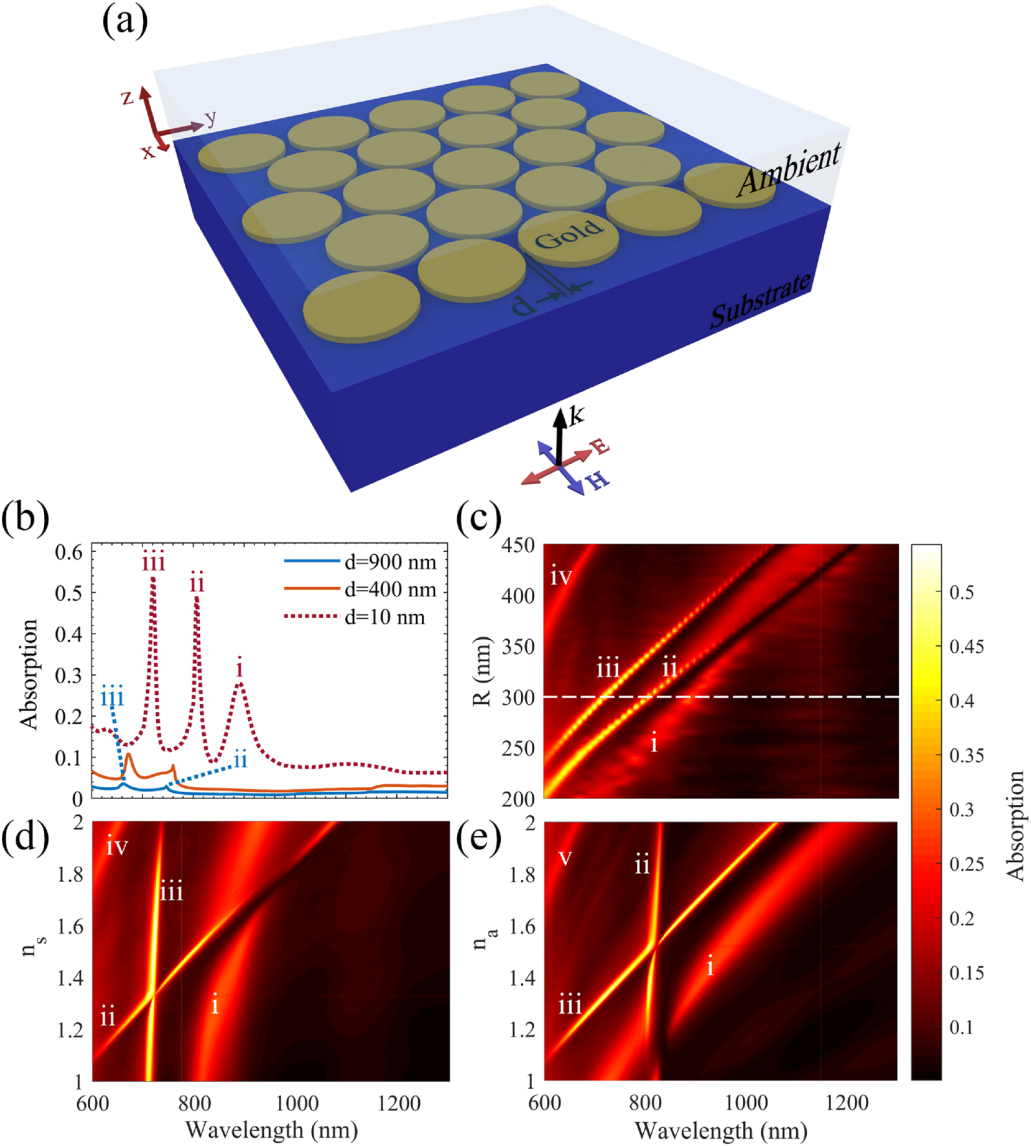
(**a**) 3D scheme of the proposed gold disc array. (**b**) Absorption spectra of the propsoed structure with *d* = 900 nm, 400 nm, and 10 nm, when *R* = 300 nm, *n*_*a*_ = 1.33, and *n*_*s*_ = 1.52. (**c**,**d**) The calculated absorption profile for closed pack gold disc array of *d* = 10 nm, versus: (**c**) *R* and *λ*_*0*_ (*n*_*a*_ = 1.33 and *n*_*s*_ = 1.52), (**d**) *n*_*s*_ and *λ*_*0*_ (*n*_*a*_ = 1.33 and *R* = 300 nm) and (**e**) *n*_*a*_ and *λ*_*0*_ (*n*_*s*_ = 1.52 and *R* = 300 nm).

As the next step, to achieve excitement of DSPMs and to enhance the intensity of LSPMs, we investigate the proposed gold disc array, which is illustrated schematically in Fig. 3a. The proposed structure is a closed packed array of gold discs with the gold thickness of 40 nm, and an interspacing distance of *d* between the gold discs*,* as shown in Fig. 3a. The proposed gold discs array allows a plasmonic behavior with less sensitivity to the illumination polarization direction, comparing with other possible array configurations^31,32^. Figure 3b demonstrates the calculated absorption spectrum for a disc array with *R* = 300 nm, and *d* = 900, 400, and 10 nm, while the refractive index of the ambient and the substrate are assumed *n*_a_ = 1.33, and *n*_s_ = 1.52, respectively. It is observable that there are two plasmonic peaks in the wavelength range below ~ 820 nm in all the presented spectra, which are marked by ii and iii, and do not shift significantly versus varying *d*. These peaks, labeled as ii and iii, are attributed to the excited LSPMs on the gold disc interfaces with the substrate and the ambient, respectively. Therefore, these peaks do not shift significantly versus varying *d,* when the disc radius is fixed. However, we expect additive superposition of the excited LSPMs on the gold discs for smaller *d* values, so that the absorption peaks intensities are increased (ii, iii) for smaller *d* values (as observed in Fig. 3b). The wavelength of plasmonic peaks ii and iii are matched with ${\lambda }_{{SP}_{1,a}}$, ${\lambda }_{{SP}_{1,s}}$ in Eq. (1). Moreover, decreasing *d* down to 10 nm, a new peak is appeared in the scattering spectrum, which is labeled as (i) in the dotted spectrum of Fig. 3b. This peak is attributed to excitement of DSPMs at the interspacing nano-slits, due to the proximity of two plasmonic edge hot spots of each disc for *d* = 10 nm. Figure 3c illustrates the absorption spectra for the indicated disc array with *d* = 10 nm, *n*_*a*_ = 1.33, *n*_*s*_ = 1.52, and for the disc readius range of *R* = 200–450 nm. In this part, curves i, ii, and iii corresspond to the discussed absorption peaks in part (b), when *R* was fixed at 300 nm (the white dashed line in part (c)). It is observable in Fig. 3c that peak (iv) is emerged in the absorption spectra for higher *R* values, and corressponds to ${\lambda }_{{SP}_{2,s}}$ in Eq. (1). Moreover, it is seen that increasing the disc radius leads to red shift of ${\lambda }_{{SP}_{M,J}}$, as expected from Eq. (1). According to Fig. 3c, we can design the disc radius to achieve the desired excitation wavelengths for our proposed plasmonic array.

Figure 3d shows the absorption spectra for the same structure for the substrate index range of *n*_*s*_ = 1–2_,_ while *n*_*a*_ = 1.33. As can be seen, peaks (ii) and (iv) very sensitive to the *n*_*s*_ value, while peaks (i) and (iii) are not varying significantly. This observation coressponds to the fact that peaks (ii) and (iv) are observed owing to excitation of the first and second plasmonic modes at the disc/substrate interface (${\lambda }_{{SP}_{1,s}}$ and ${\lambda }_{{SP}_{2,s}}$), respectively. Then, we have investigated the variations of the absorption spectra versus varying the ambient index in the range of *n*_*a*_ = 1–2 in Fig. 3e, while *n*_*s*_ = 1.52. Figure 3e demonstrates that plasmonic peaks labled as (i), (iii), and (v) are sensitive to varying *n*_*a*_, while peak (ii) does not change significantly. Thus, the proposed structure can also behave as a high sensitive plasmonic sensor for detecting the trapped target particles, especially in the LSPM trapping state. Peaks (iii) and (v) in this figure are attributed to the first and the second SPP modes at the disc/ambiet interface (${\lambda }_{{SP}_{1,a}}$ and ${\lambda }_{{SP}_{2,a}}$), respectively. The other point here is that peak (i), which is attributed to excitement of DSPMs in the disc interspacing nano-slits, is significantly depended on the ambient index (as shown in part (e)), however, it is approximately non-depended on the substrate index (as shown in part (d)). This ambient sensitivity of peak (i) (DSPMs) makes sense because the interspacing cavity is filled with the ambient in our structure.

**Figure 4:**
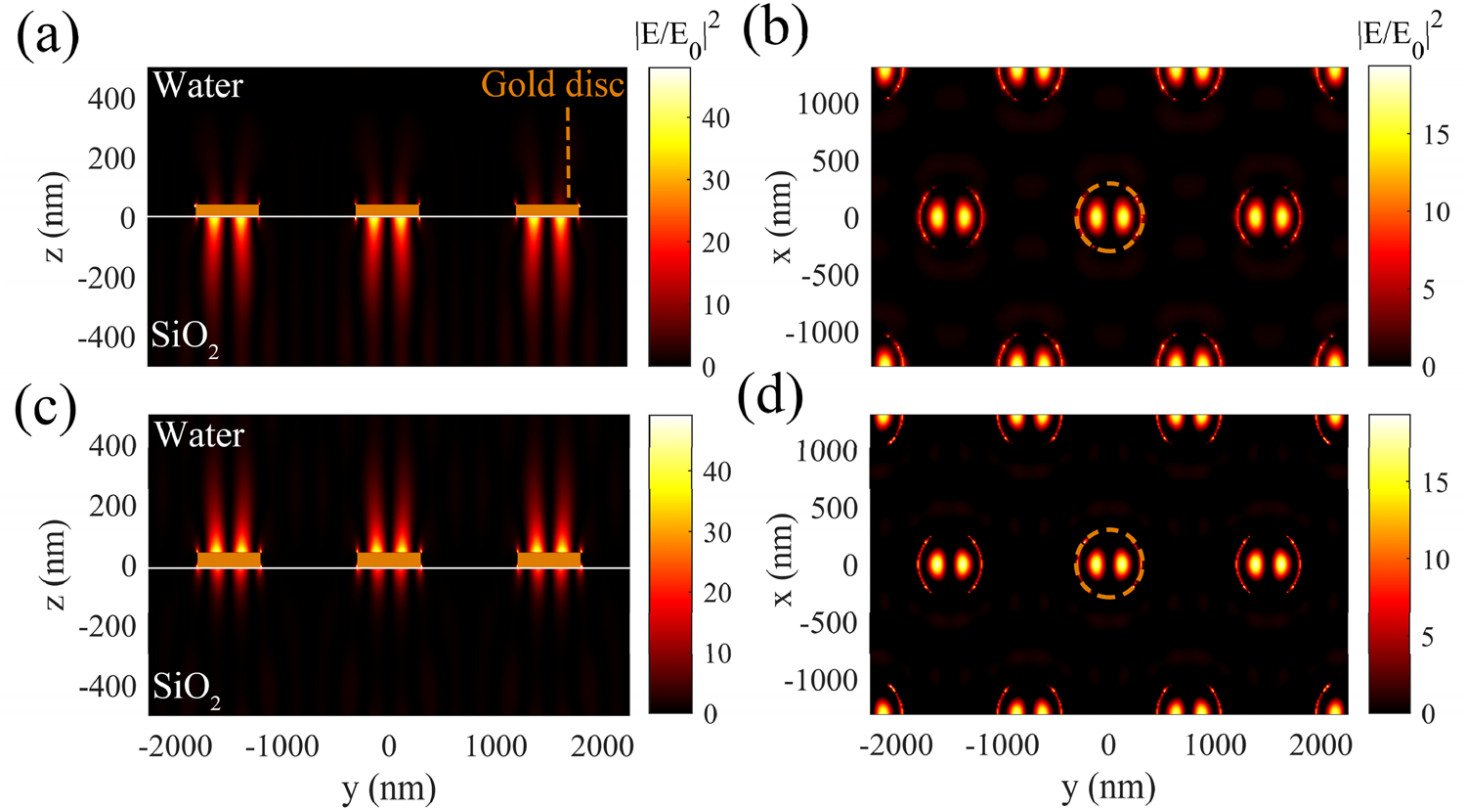
(**a**) the cross section, and (**b**) top views (at *z* = − 10 nm) of the normalized field intensity distribution corresponding to peak (ii) of the blue spectrum in Fig. 3b. (**c**) The cross section, and (**d**) the op views (at *z* = 10 nm) of the normalized field intensity distribution, corresponding to peak (iii) of the blue spectrum in Fig. 3b. The orange solid rectangles in parts (**a**) and (**c**), and the dashed circles in parts (**b**) and (**d**) represent the cross section and top views of the gold discs, respectively.

For more clarification, we have plotted field distributions on the disc arrays, corressponding to plasmonic peaks in Fig. 3b. Parts (a) and (b) in Fig. 4 show the cross section and the top (at *z* = − 10 nm) views of the normalized electric field distribution, coressponding to peak (ii) of the blue spectrum in Fig. 3b, when *R* = 300 nm, and *d* = 900 nm. It is evidently observed that in these figures LSPMs are excited at the disc/SiO_2_ interface, when the incident wavelength is ${{\varvec{\lambda}}}_{{{\varvec{S}}{\varvec{P}}}_{1,{\varvec{s}}}}$.

Figure 4c,d show the cross and the top (at *z* = 50 nm) views of the normalized electric field distributions at the incident wavelength of ${\lambda }_{{SP}_{1,a}}$, coressponding to peak (iii) of the blue spectrum in Fig. 3b, when *R* = 300 nm, and *d* = 900 nm. It is clear in these parts that LSPMs are excited at the disc/ambient (water) interface at ${\lambda }_{{SP}_{1,a}}$. It is notable that ${\lambda }_{{SP}_{1,s}}>{\lambda }_{{SP}_{1,a}}$, due to *n*_*s*_ > *n*_*a*_, and according to Eq. (1). Emergence of the first LSPM modes with one wavelength along the incident polarization, correspponding to the gold interfaces with the ambient and substrate, are observable in the cross section and top view field distributions. For more clarification, one of the gold discs is shown by dashed circles in both top views of the field distributions in parts (b) and (d) of Fig. 4. Evidentaly, at higher-order LSPM modes, higher factors of the wavelengths should be emerged on the surface of the nano-discs.

**Figure 5:**
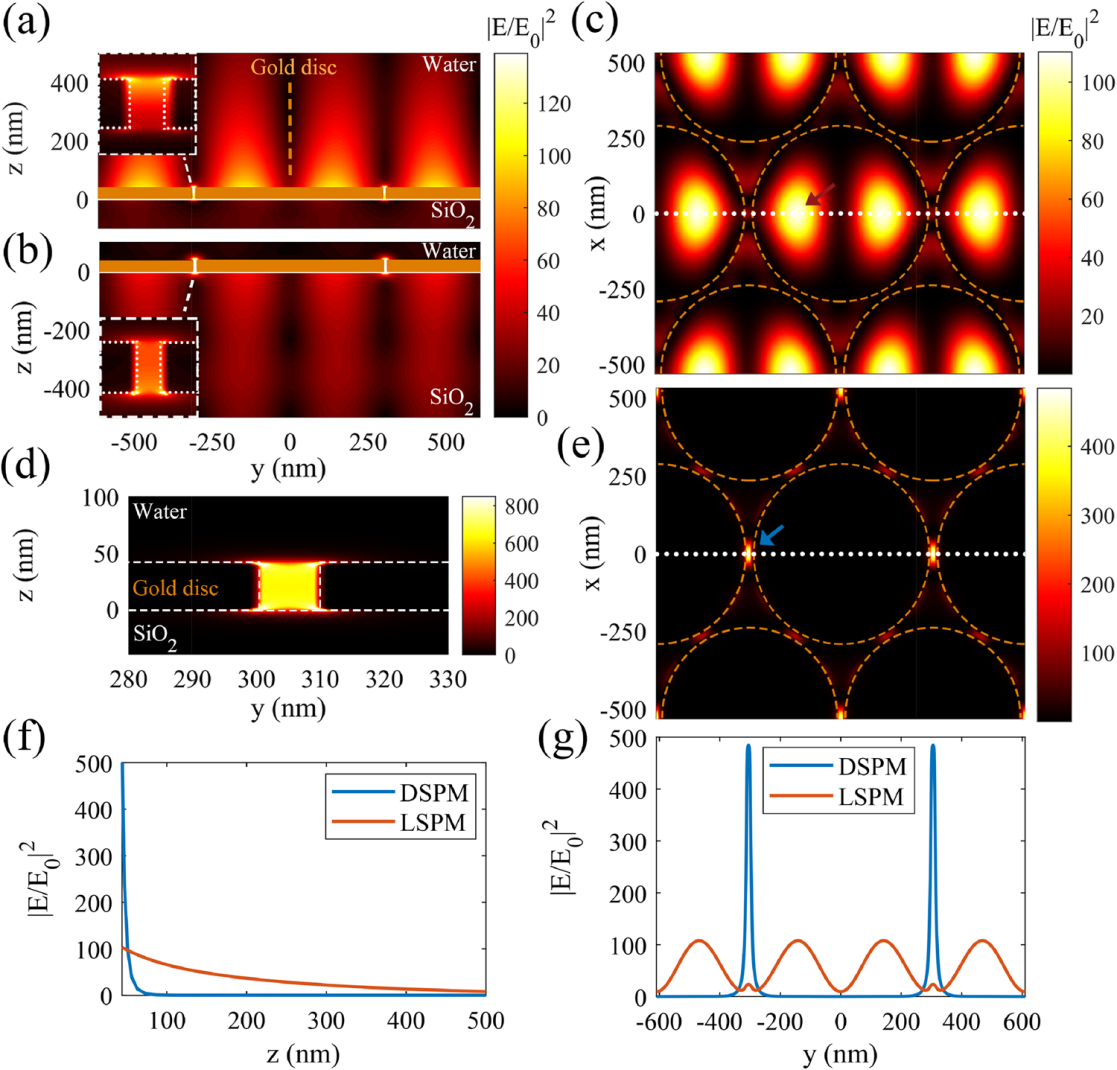
(**a**,**b**) The cross section views of the field intensity distribution for LSPMs, corresponding to peaks (ii) and (iii) of the red dotted spectrum in Fig. 3b. (**c**) The top view (at *z* = 50 nm) of the normalized field intensity distribution corresponding to peak (iii) of the red spectrum in Fig. 3b. (**d**,**e**) The cross section and top views (at *z* = 50 nm) of the normalized field distribution corresponding to peak (i) of the red dotted spectrum in Fig. 3b. (**f**) Variations of the normalized field intensities of the LSPM and DSPM along the *z* direction at the *x*–*y* position, where the relating field intensities are maximized (shown by the red and blue arrows in (**c**,**e**)). (**g**) Variations of the normalized field intensities of the LSPM and DSPM along the white dashed line in (**c**,**e**), at *z* = 50 nm. The orange solid rectangles in parts (**a**,**b**), and the dashed circles in parts (**c**,**e**) represent the gold discs.

Then, we have investigated our proposed structure with *d* = 10 nm, and plotted the field distributions from the side and top views at the plasmonic peaks of the corressponding spectrum (red dotted spectrum in Fig. 3b) in Fig. 5. Figure 5a,b illustrate the cross views of the normalized field intensity, relating to peaks (iii) and (ii) of the red dotted spectrum in Fig. 3b, respectively. In part (a), it is evident that LSPMs are excited on the gold discs at the gold/ambient (water) interface, while part (b) shows the excited LSPMs between the gold and the substrate (SiO_2_). Gold discs are shown by the orange solid rectangles and dashed circles in the cross section and top views of the field distributions in this figure respectively. The insets in parts (a) and (b) show the magnified views of the field distribution at the disc edges. Higher field intensity at the up edge of the nano-slit is in agreement with the excitation of the LSPMs at the top surface of gold edge (disc/ambient interface) in Fig. 5a. Similarly, higher intensity of the field at the bottom edge of the nano-slit confirms the excitation of LSPMs at the disc/substrate interface in Fig. 5b. Figure 5c illustrates the top view field distribution of the described disc array at *z* = 50 nm, relating to peak (iii) in the red dotted spectrum in Fig. 3b. The plasmonic fields shown in parts (a) and (c) indicate the excitation of the first-order LSPM at the disc/water interface (${\lambda }_{{SP}_{1,a}}$), while part (b) indicates the excited first mode of LSPMs at the disc/substrtae (${\lambda }_{{SP}_{1,s}}$). Figure 5d,e show the cross and top (at *z* = 50 nm) views of the normalized field distribution of the gold disc array, corressponding to peak (i) in the red dotted spectrum of Fig. 3b. These figures show excitation of DSPMs between the neighboring nano-discs with *d* = 10 nm, due to proximity of the neighboring edge plasmonic hot spots. Figure 5f,g compare the variations of the field intensities of LSPM (red curves) and DSPM (blue curves) modes along the *z* and *y* directions, respectively. Part (f) shows the *z* direction variations of the LSPM and DSPM at their corressponding maximum field positions in the *x*–*y* plane, shown by red and blue dots in Fig. 5c,e, respectively. It can be observed in part (f) that DCPMs show a lower penetration depth in water, in comparison with LSPMs. However, the mode intensity at the surface of gold discs is higher for DSPMs, as compared with LSPMs. Thus, excitation of LSPMs leads to higher penetration depth, allowing to attract the target particles from further distances. Then, switching the incident wavelength to excite DSPMs, results in high gradient intensity in the vicinity of the disc surface, which allows strong trapping of small target particles. Figure 5g illustrates variation of the LSPM and DSPM intensities versus the *y* direction along the white dashed lines in Fig. 5c,e, respectively. This figure proves that the maximum intensity of DSPMs is about 5 times higher than the maximum intensity of LSPMs, at the same incident power. Moreoever, it is highlighted that the DSPMs are maximized at the nano-slit between the discs, while LSPMs are maximized on the discs.

### Investigation and comparison of plasmonic force based on LSPMs and DSPMs

**Figure 6:**
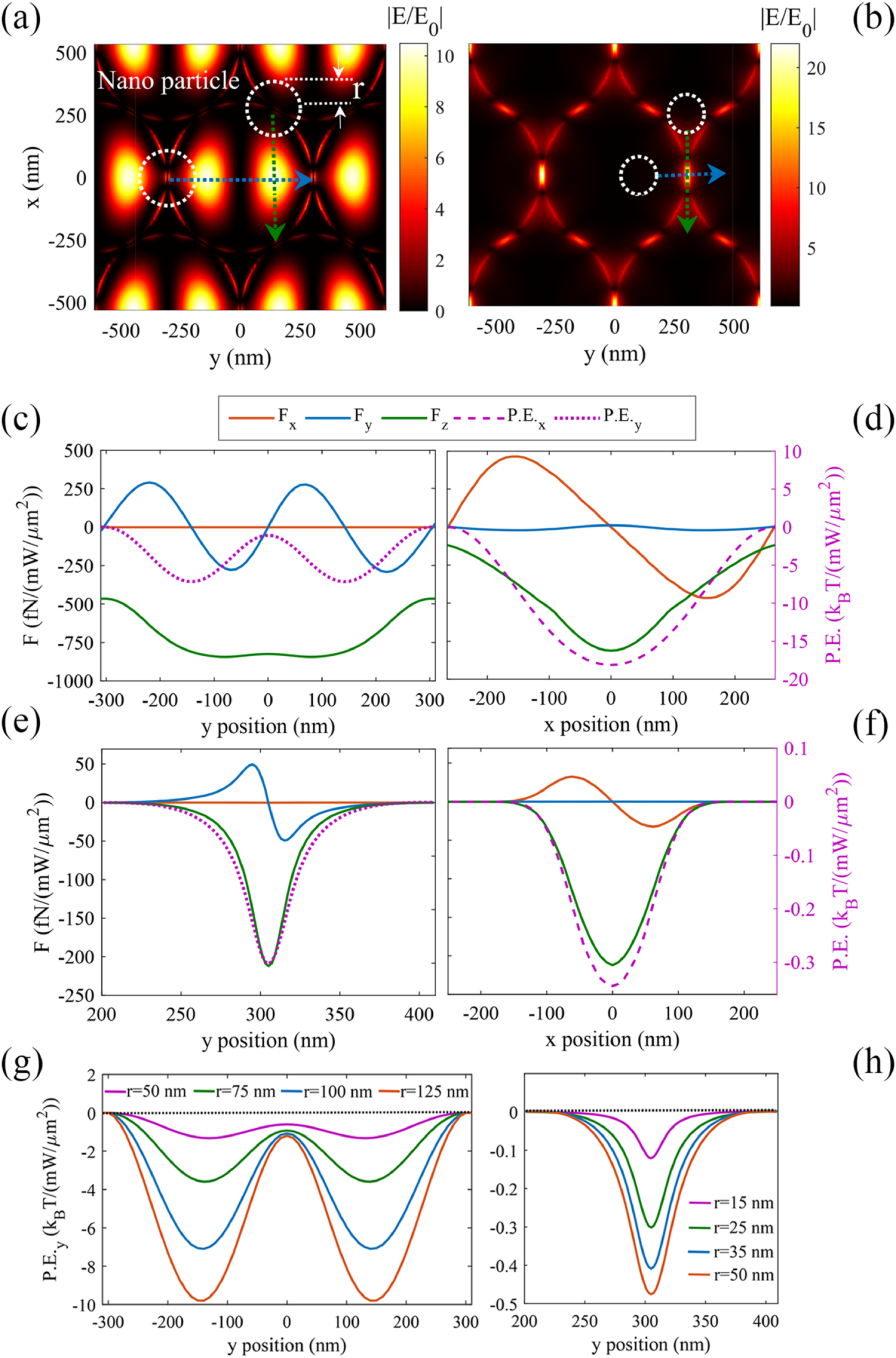
The investigated particle motion directions with respect to the plasmonic field distributions for (**a**) LSPM, and (**b**) DSPM. The white dotted circles show the particles, while the blue and green dotted arrows show the particle motions along the *y* and *x* directions, respectively. The exerted plasmonic force components and the resulting potential energies are presented versus the particle position along the *x* and *y* axis, for polystyrene spheres with (**c**,**d**) *r* = 100 nm and LSPM excitation, and (**e**,**f**) *r* = 25 nm and DSPM excitation, at fixed *z* = 50 nm. (**g**) LSPM, and (**h**) DSPM potential energies for polystyrene particles with different radii, when the particle is moved along the *y* direction at *z* = 50 and *x* = 0 nm.

Here, we investigate the plasmonic forces exerted to the target particles. It is notable all the forces and potentials obtained here have been normalized to the intensity of input sources. First, we present the plasmonic forces exerted on the polystyrene nanoparticle with a refractive index of 1.5717. Figure 6a shows the absolute field of the first LSPM, excited at the incident wavelength of ${\lambda }_{{SP}_{1,a}}$, wherein the dotted blue and green arrows show the investigated moving directions of the target polystyrene particle, in order to investigate the maximum interaction of the particle with the excited plasmonic field. Figure 6b indicates the absolute field of the excited DSPM, wherein the blue and green dotted arrows show the investigated y and x moving directions of the polystyrene particle. In both parts, the white dotted circles represent the target particles, and the vertical position of the particles are assumed *z* = 50 nm (at a distance of about 10 nm from the gold surface). Figure 6c,d (Fig. (e,f)) show different components of the calculated plasmonic force relating to the LSPMs (DSPMs), exerted on the polystyrene nanoparticle with radius of *r* = 100 (25) nm, beside the resulted potential energies along *y* (dotted curves) and *x* (dashed curves) directions, respectively.

Figure 6c proves that the minimum of the potential energy along *y* direction occurs at *y* = 145 nm, wherein the target particles are trapped. To investigate the trapping behavior along the *x* direction, we have assumed *y* = 145 nm, and *z* = 50 nm, calculated the plasmonic forces and the resulted potential energies (Fig. 6d). Moreover, LSPMs lead to a wider trapping site, while DSPMs leads to a narrower trapping site for the target particles. In other words, at the vicinity of the gold surface, trapping behavior of DSPMs is spatially more confined, as compared with LSPMs. According to Fig. 5c,e, it is clear that the field distributions is wider along the x direction, as compared with the y direction due to the incident polarization direction. Therefore, the achieved potential depth is wider along x direction, comparing with that along the y direction in Fig. 6. As expected, considering the presented potential energies of the LSPMs and DSPMs, we find that a deeper potential depth and a stronger trapping behavior are achieved along the *x* direction, or the incident polarization. Considering this fact, to study the trapping capability of the structure, we calculate the force and potential depth along the y direction, and find the minumum required incident power for stable trapping of the particles along y direction in the rest of figures. To investigate the effect of particle size on the applied potential energy, we have plotted the potential energies of LSPMs and DSPMs for particles with *r* = 50 nm, 75 nm, 100 nm, and 125 nm in Fig. 6g, and particles with *r* = 15 nm, 25 nm, 35 nm, and 50 nm in Fig. 6h, all at *z* = 50 nm, respectively. It can be seen that LSPMs with a source intensity of 1 mW µm^−1^ provides a potential well of 10 *k*_*B*_*T* for a particle of *r* = 125 nm. These results prove that larger particles show larger scattering cross-sections and larger plasmonic forces, and deeper potential wells, consequently^13^. Moreover, it can be observed in Fig. 6g,h that DSPMs allow trapping of particles smaller than 34 nm, due to the highly confined plasmonic fields.

**Figure 7:**
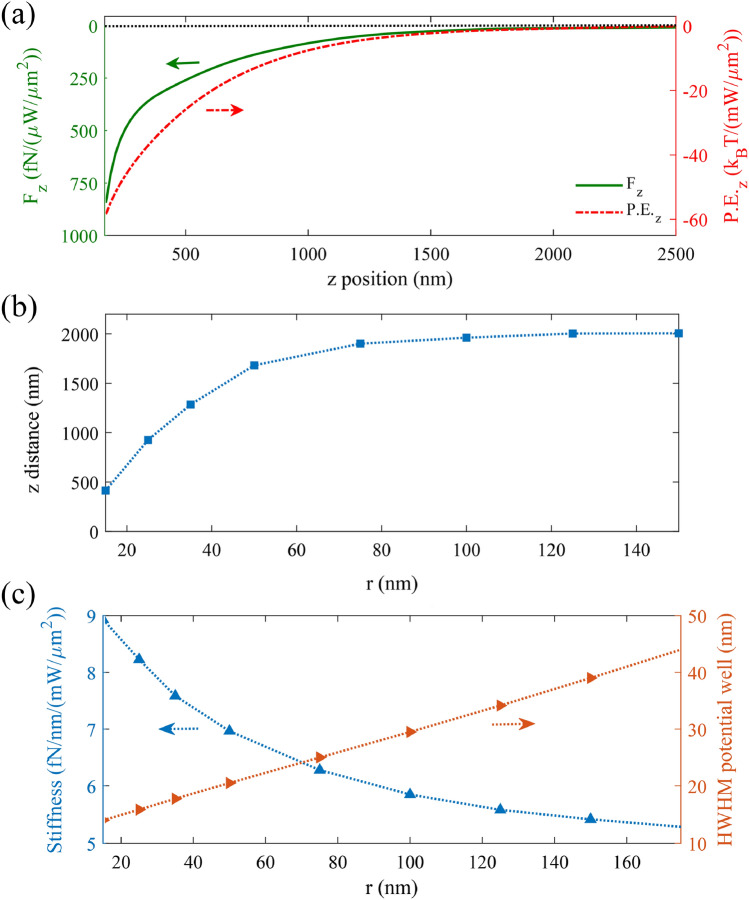
(**a**) The left and right axes show the z component of the LSPM force and the resulting potential, versus the *z* position of the particle with *r* = 100 nm, at the *x*–*y* position wherein the potential energy is minimized (*x* = 0, *y* = 145 nm). (**b**) Variation of the LSPM trapping height, wherein *P.E.*_*z*_ equals 10*k*_*B*_*T*, versus varying the particle radius. The incident power has been assumed 50 mW μm^−2^. (**c**) DSPM related trapping stiffness along *y* direction (*s*_*y*_) (blue curve), and HWHM (red curve) versus the radius of the nanoparticle.

Then, we investigate the *z* component of the LSPMs-induced gradient force versus varying the *z* position of the target particle. For this purpose, we have moved a polystyrene particle along the *z*-axis at the *x*–*y* trapping position at *x* = 0, *y* = 145 nm, wherein the LSPM intensity is maximized and the resulting potential energy is minimized. Figure 7a indicates the *z* component of the plasmonic force exerted to a polystyrene particle with radius of 100 nm, and the resulting potential depth, versus varying the *z* position of the particle up to 2.5 μm. We define the effective trapping height as the *z* value, wherein potential depth exceeds 10 *k*_*B*_*T* for the source intensity of 50 mW µm^−2^. Figure 7b illustrates that the calculated effective trapping height is increased for larger particles, and it extends to more than 2 μm for target particles with the radius larger than 100 nm. According to Fig. 6e, the force is linearly related to the deflection of the particle from its equilibrium trapping point, so that we can extract the trapping stiffness along the *y* direction for the DSPM trapping by using Eq. (5). In Fig. 7c, the left and right axes display the variation of the trapping stiffness (*s*_y_) and half width half maximum (HWHM) for the LSPM trapping, versus the particle radius. It is observed that by increasing the radius of the nanoparticle from 15 to 175 nm, *s*_y_ decreases, while HWHM increases. For instance, Fig. 7c reveals that we can achieve *s*_y_ = 9 fN nm^−1^/(mW µm^−2^), and HWHM = 14 nm for a nanoparticle with *r* = 15 nm by DSPMs. As observed in Fig. 7c, the plasmonic force and the relating trapping stiffness does not change linearly versus the particle radius^13^, but their relation depends on the effective spatial overlap between the polystyrene particle and the plasmonic field. On the other hand, the allowed displacement variations of the trapped particle can be expressed by HWHM, which is shown to have a linear relation with the particle size (Fig. 7c). Dividing the plasmonic force to the HWHM in the investigated range of particle radius (15–175 nm), we have shown that the trapping stiffness is increased by decreasing the particle size, while the increasing trend is amplified for smaller particles. This increasing trend is attributed to the 10-nm gap in our structure and the relating highly confined DSPM field, leading to the maximum spatial overlap between the particle and the plasmonic field, when the particle size is comparable with the gap size. In other words the plasmonic interaction between the DSPM and the dielectric particle, and the resulting plasmonic force is maximized for particles sizes around the gap size. Accordingly, we expect decreasing trapping stiffness versus decreasing the particle radius smaller than about 5 nm. However, we avoid expanding our classical simulations to particle radii smaller than about 15 nm here, wherein quantum effects become significant gradually.

**Figure 8:**
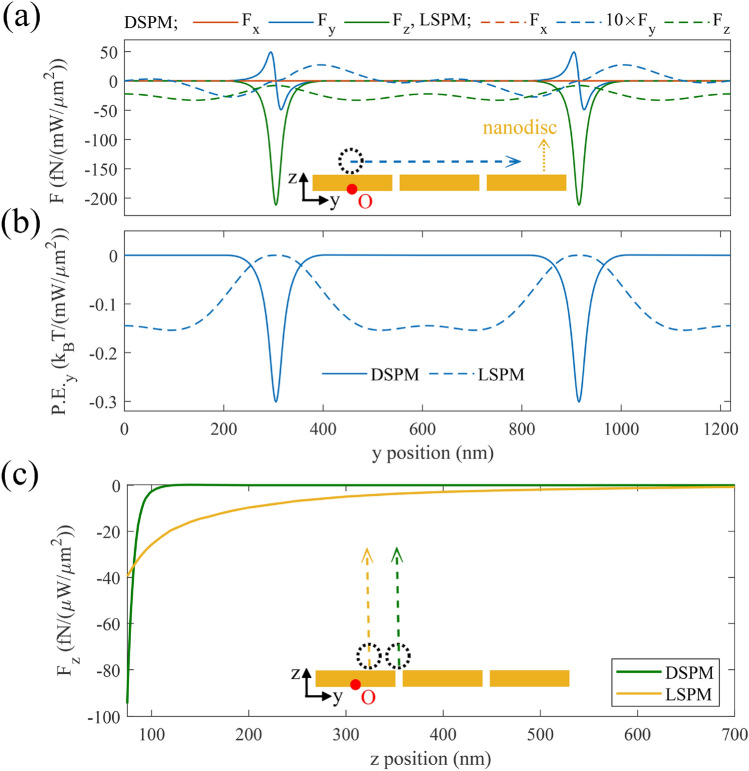
(**a**) The exerted force, and (**b**) the potential energy for a polystyrene particle with *r* = 25 nm along the *y* direction (blue dashed arrow) at *x* = 0, and *z* = 50 nm relating to the LSPM (dashed curves), and DSPM (solid curves) trapping modes. (**c**) Variations of *F*_*z*_ of the LSPM (orange), and DSPM (green) for polystyrene particles at their corresponding trapping equilibrium *x*–*y* positions, versus varying *z* position. The insets show the schematic cross section of the gold discs, and the dashed arrows show the particles moving paths. In the inset of part (**c**) orange and green dashed arrows show the particle movement along the *z* direction, corresponding to the LSPMs and DSPMs, respectively.

Now, to determine the practical benefits of both trapping mode, we compare forces and potential wells exerted to a polystyrene particle with *r* = 25 nm for both LSPMs and DSPMs. The inset in Fig. 8a shows the cross view of the investigated particle's motion above the proposed gold discs, schematically. Here, the particle is moved along *y* directions with a vertical distance of 10 nm from the gold surface (*z* = 50 nm). Parts (a) and (b) in this figure show different components of the exerted plasmonic force, and the resulting potential depth along *y* direction, respectively. Figure 8a, such as Fig. 6c–f, displays that LSPM gradient force is exerted to the target particle, almost all over the surface, in contrary with the DSPM localized gradient force. Hence, LSPM trapping can be beneficial for the initial trapping of the target particles, regardless of their initial *x*–*y* position. Furthermore, this part reveals that *F*_*z*_ for DSPMs is achieved about 5 times higher than that for LSPMs, which is in accordance with the corresponding mode intensities in Fig. 5g. Therefore, to hold the particles in the vicinity of the gold surface we can use DSPM trapping with lower power consumption. Part (b) reveals that DSPMs show deeper potential wells and stronger trapping behavior for small particles. So that, It can be found from this figure that a target nanoparticle with a *r* = 25 nm can be trapped by LSPMs, when the incident wave length is 720 nm, and the incident power is 64.93 mW µm^−2^. Otherwise, this particle can be trapped by DSPMs, when the incident wavelength is 900 nm and the incident power is 33.19 mW µm^−2^. Thus, we find that DSPMs can lead to the particle trapping with less power consumption, equal to half of the power needed for LSPMs trapping. Additionally, the investigated target particle is trapped on the nano-slit spacing between the discs for DSPMs, while it is trapped on the gold disc for LSPMs. In other words, it is quite observed that the DSPM trapping sites are very localized and isolated from each other, as compared with the LSPM trapping sites. Therefore, we can benefit from DSPMs for trapping target quantum dots in separate and isolated positions. Figure 8c indicates variations of *F*_*z*_ versus varying *z* position of the target particle, which is trapped by LSPMs (*x* = 0, *y* = 145 nm) and DSPMs (*x* = 0, *y* = 305 nm). In this figure the long range and weak force behavior of the gradient force is evidently observable for LSPMs, in contrast to the short range and strong force behavior of the DSPMs. These different trapping features are attributed to the generally larger penetration depth of SPPs (such as LSPMs) than that of LSPs (such as DSPMs).

Benefiting from discussed LSPMs and DSPMs in the proposed plasmonic structure, allows attracting quantum dots from the microfluidic channels toward the plasmonic structure, then by switching the incident wave length with a low optical power, they can be trapped strongly. Moreover, we have the apportunity of trapping the particles by LSPMs to achieve more degree of spatial delocalization, which allows more interaction between the trapped particles. Otherwise, trapping the particles by DSPMs leads to higher localization of the trapped particles at separate trapping sites, which reduces the possible interaction between the particles. In other words, as shown in Fig. 8b, LSPMs lead to trapping zones with interspacing of about 190 nm, while DSPMs lead to trapping sites with interspacing of about 610 nm. This reveals that the proposed plasmonic tweezers can serve as an appropriate tool for studying the interactions between the target nanoparticles.

**Figure 9:**
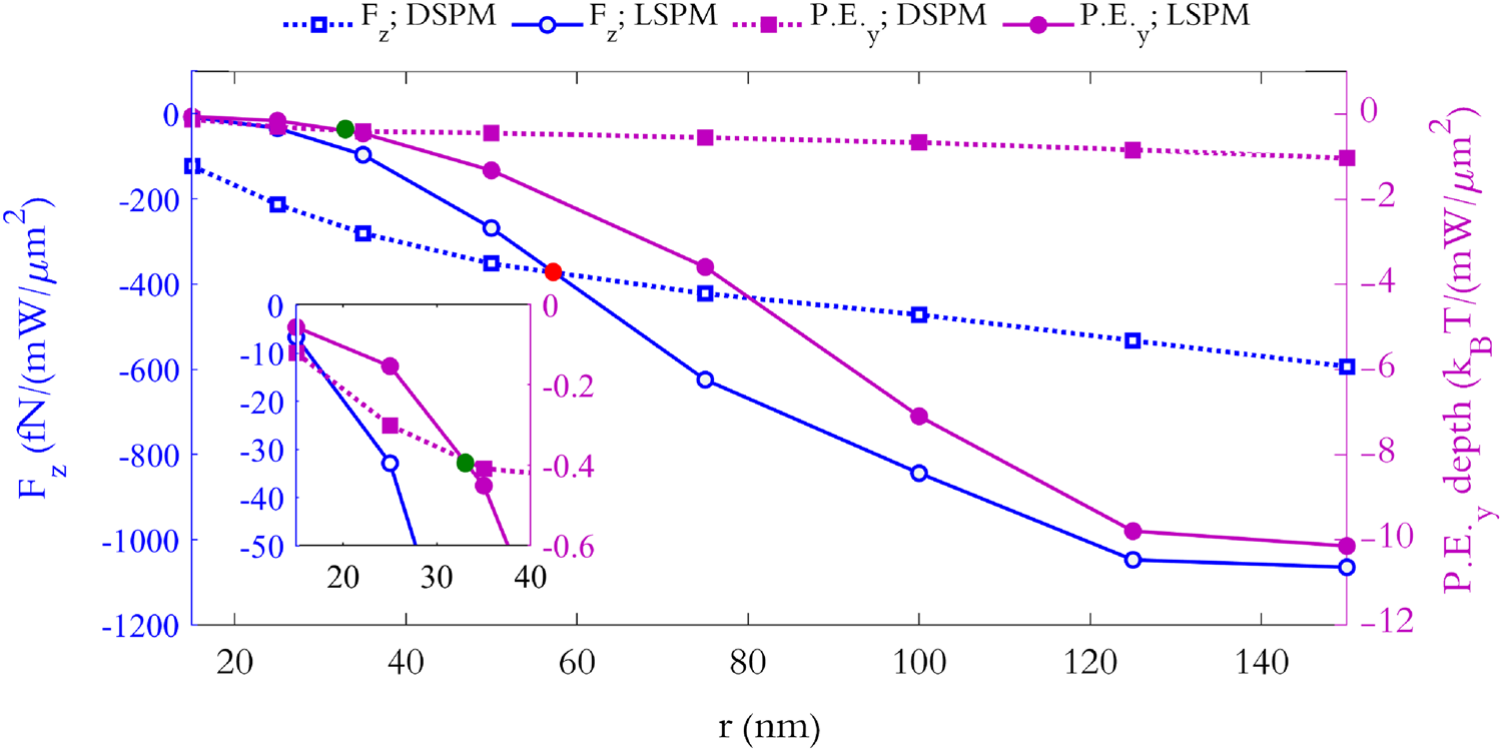
The left green axis presents the maximum exerted *F*_*z*_, at *x* = 0 nm and *y* = 300 nm for LSPM, and at *x* = 0, *y* = 145 nm for DSPM, versus different radii of the particle. The particle is assumed at a vertical distance of 10 nm from the nano-discs surface. The purple right axis represents the depth of the potential well along the y direction, exerted to particles with *r* = 15–150 nm. The inset exhibits the magnified view of the LSPM (solid purple) and DSPM (dotted purple) *P.E.*_*y*_ depth variations around their intersection, marked by blue dot. The red dot highlights the intersection of the *F*_*Z*_ curves relating to LSPM and DSPM trapping modes.

Now, to elaborate the trapping functionality, we study the minimum required power for stable trapping of the particles in each mode. The left blue axis of Fig. 9 shows the exerted *F*_*z*_ to the polystyrene particles with different *r* values, for both the LSPM and DSPM trappings, at *z* = 50 nm and the corresponding *x*–*y* trapping positions. Moreover, the right purple axis in this figure indicates the normalized potential depth along the *y* direction for particles with different radii, at *x* = 0 nm and *z* = 50, for both LSPM and DSPM trappings. The green dot on the purple curves (*P.E.*_*y*_ depth curves), which is better shown in the magnified view in the inset, indicates that for nanoparticles with *r* < 34 nm, In addition to *F*_*z*_, *P.E.*_*y*_ depth created by DSPM is greater than that of LSPM. In other words, DSPMs is the dominant trap with less power consumption, for particles with *r* < 34. The red dot on the blue curves (*F*_*z*_ curves) indicates that the maximum *F*_*z*_ value, exerted by LSPMs is greater than that of the DSPMs for *r* > 60 nm. In other words, for nanoparticles with *r* > 60 nm, LSPMs is more suitable for low power trapping. However, for *r* < 34 nm DSPMs is more suitable for low power trapping. moreover, for 34 < *r* < 60 nm, LSPMs traps particles on the *x*–*y* plane with less power consumption than DSPMs, owing to their deeper potential well, and conversely, DSPMs holds the trapped particles near the surface with Less power consumption than LSPMs owing to their stronger *F*_*z*_.

Finally, we study the thermal issues in both operation modes of the proposed plasmonic tweezers briefly. It should be considered that plasmonic structures such as nano-holes in a gold layer, benefiting from high in-plane thermal conductivity, lead to less heat generation than the gold nano-discs in despite of their high absorption^42^. Considering the 10-nm water-filled gaps in our proposed closed packed gold disc array, we expect the in-plane thermal conductivity can limit and diminish the thermal issues, similarly. To elaborate the thermal issues in our structure, first it should be noted that the generated heat in microfluidic channels creates three types of forces. The first is the stochastic Langevin force, causing Brownian motion of the particle, and is achieved by $\langle {\mathrm{F}}_{\mathrm{th }}\left(\mathrm{t}\right){\mathrm{F}}_{\mathrm{th }}\left({\mathrm{t}}^{\mathrm{^{\prime}}}\right)\rangle =2{\mathrm{k}}_{\mathrm{B}}\mathrm{T\gamma \delta }\left(\mathrm{t}-{\mathrm{t}}^{\mathrm{^{\prime}}}\right),$ wherein, $\gamma$*,* *T*, and ${k}_{B}$ are the drag coefficient, the fluid temperature, and the Boltzmann constant, respectively. The drag coefficient is calculated by $\upgamma =6\mathrm{\pi \eta r}$, wherein *η* is the fluid viscosity. The delta function δ(t–t′) shows the independent characteristic of the stochastic Langevin forces at two distinct times^24,43^. The second force is the Stokes’ drag force ${\mathrm{F}}_{\mathrm{D}}=\mathrm{\gamma v}$, wherein *v* is the fluidic convection velocity vector, originating from the temperature gradient, induced by the plasmonic hot spots in our plasmonic tweezers. This fluidic convection can also cause displacement of the trapped particles and affect plasmonic trapping^44^. The third force is the thermophoretic force ${\mathrm{F}}_{\mathrm{T}}=-\upgamma {\mathrm{v}}_{\mathrm{T}}$, originating from the particle drift along the temperature gradient ($\nabla T$). The steady state thermophoretic velocity (${v}_{T}$) is calculated by ${\mathrm{v}}_{\mathrm{T}}=-{\mathrm{D}}_{\mathrm{T}}\nabla \mathrm{T}$, wherein *D*_*T*_ is the thermophoretic mobility^24,44,45^. The thermophoretic mobility of polystyrene particles immersed in water with different radii have been measured and reported in a range of 1 < *D*_*T*_ < 10 μm^2^ s^−1^ K^−1^^46,47^.

**Figure 10:**
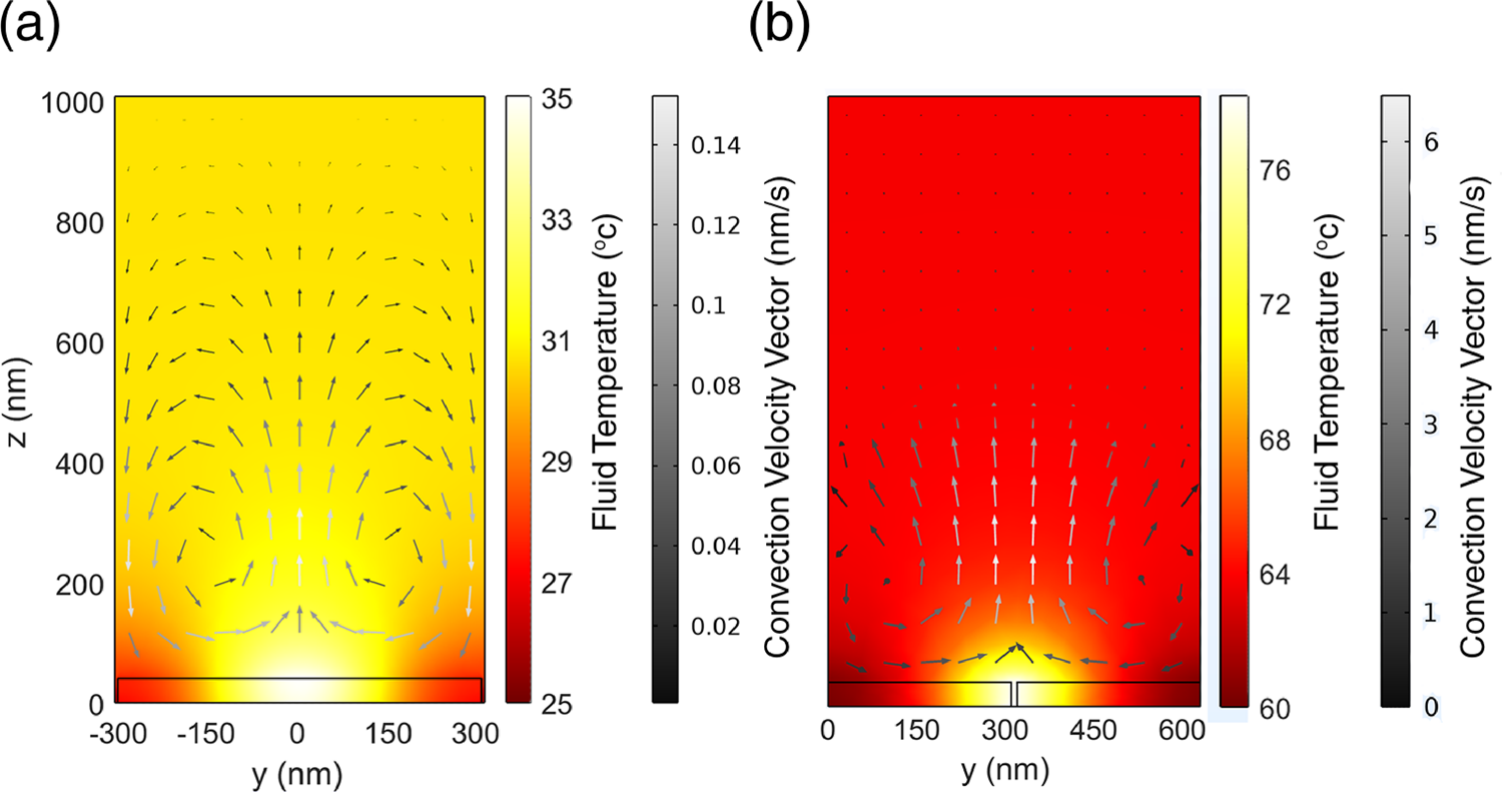
Temperature distribution and the fluidic convection velocity vectors (arrows) in the surrounding aqua medium, when (**a**) LSPMs, and (**b**) DSPMs are excited by the incident power densities of 7.7 mW μm^−2^ and 83 mW μm^−2^, respectively.

We have plotted the temperature distribution at the *y–z* plane (*x* = 0), and the resulting fluidic convection velocity vectors in the proposed plasmonic tweezers for both LSPMs (Fig. 10a) and DSPMs (Fig. 10b), while the input source intensities are 7.7 mW μm^−2^ and 83 mW μm^−2^, respectively. It is notable that we have used finite element method and assumed *T* = 20 °C at the top/down boundaries, in our thermal simulations. Moreover, incident power densities are assumed equal to the maximum applied power densities, so that they can provide the required power to trap the smallest investigated polystyrene particles with *r* = 15 nm and 50 nm in each LSPM and DSPM mode (Fig. 6g,h). It can be observed in Fig. 10 that higher temperature and temperature gradient is achieved for DSPMs in the surrounding environment, due to the higher field confinement in DSPMs than the LSPMs. Figure 10 indicates that the maximum temperature is achieved about 35 °C (78 °C) for the LSPMs (DSPMs) excitations, which is comparable with other reported plasmonic tweezers based on LSPs in metallic structures^48^. According to the achieved temperature distribution and the consequent fluidic convection velocities in Fig. 10, the maximum thermal-induced forces are calculated as: *F*_*th*_ ≈ 7 fN, *F*_*D*_ ≈ 1.3 × 10^–4^ fN, *F*_*T*_ ≈ 13 fN for LSPMs, and *F*_*th*_ ≈ 24 fN, *F*_*D*_ ≈ 1.65 × 10^–3^ fN, *F*_*T*_ ≈ 4.5 fN for DSPMs. It should be noted that at the same conditions, the *y*-component of the plasmonic force has been calculated 385 fN for the LSPMs, and 2 pN for the DSPMs. Hence, it can be concluded that the thermal-induced forces are negligible as compared with the exerted plasmonic forces, and thermal issues cannot interfere with the plasmonic trapping operation of the designed plasmonic tweezers considerably.

## Conclusion

In summary, we proposed a periodic array of closed pack gold nano-discs on glass as an efficient and controllable plasmonic tweezers that benefits from dual plasmonic trapping modes with promising and complementary functionalities. The designed plasmonic tweezers with disc radius of 300 nm and interspacing of 10 nm can be excited in both LSPM and DSPM modes by switching the incident wavelength from 720 to 900 nm. We showed that each trapping mode offers some promising advantages, which can be used in complementary of the behavior of the other trapping mode. LSPMs have proved a longer penetration depth in the water and a consequent long range trapping capability, beside a lower gradient force magnitude. Moreover, LSPM trapping leads to wider lateral distribution for the in-plane position of the trapped particle, which allows proximity of the trapped particles in the *x*–*y* plane, and investigation of the particle–particle interactions. On the other hand, DSPMs have shown localized plasmonic fields at the gap spacing between the gold discs with very low penetration depth in water. DSPM trapping leads to a short range trapping behavior with laterally localized trapping sites, leading to isolated arrangement of the trapped particles. Moreover, DSPMs benefit from a high gradient force and high trapping stiffness, and so that allows trapping of small target nanoparticles with a radius of less than 34 nm. We can utilize LSPM trapping attract the target nanoparticles with a radius of 100 nm toward the gold surface from a vertical distance of about 2 µm. Moreover, we proved a stable LSPM trapping (considering the trapping criteria of 10 k_B_T) of polystyrene particles with radius of 125 nm, and a half width half maximum (HWHM) of 85 nm by incident intensity of 1 mW µm^−2^. The achieved wide HWHM of the LSPM trapping allows studying the interactions between the trapped particles, due to their proximity. By switching the incident wavelength, we have the ability to trap small nanoparticles with a radius of 15 nm in DSPM trapping mode, with a narrow HWHM of 14 nm and a high trapping stiffness of 9 fN nm^−1^/(mW µm^−2^). Moreover, the maximum generated temperature due to the plasmon-induced hot spots is calculated about 35 °C (78 °C) for LSPMs (DSPMs) with incident power density of 7.7 mW μm^−2^ (83 mW μm^−2^), and the resulting thermal-induced forces are negligible as compared with the exerted plasmonic forces in our simulations. Thus, the proposed dual mode plasmonic tweezers has revealed promising and complementary functionalities in each mode, suitable for different applications such as efficient trapping, sorting, and separation of target nanoparticles, or investigating particle–particle interactions. Overall, we believe that the proposed novel design and the discussed systematic results present a new insight for realizing efficient and dual operation-mode plasmonic tweezers with complementary operation, suitable for studying target nanoparticles.
